# Cooperative housing under a grant-of-use in Catalonia and health: pre-post analysis

**DOI:** 10.1186/s12889-024-19214-1

**Published:** 2024-07-09

**Authors:** Alexia Reyes, Irene Macaya Munell, Carme Borrell, Joao Pedro Carmezim Correia, Ana Fernández, Constanza Vásquez-Vera, Katherine Pérez, Juli Carrere, Lali Daví, Ana M. Novoa

**Affiliations:** 1 Agència de Salut Pública de Barcelona (ASPB), Pl. Lesseps 1, Barcelona, 08023 Spain; 2 Department of Experimental and Health Sciences, Universitat Pompeu Fabra, Doctor Aiguader 88, Barcelona, 08003 Spain; 3 Institut de Recerca Sant Pau (IR SANT PAU), C. Sant Quintí 77, Barcelona, 08041 Spain; 4 CIBER Epidemiología y Salud Pública (CIBERESP), Av. Monforte de Lemos 3-5, Pabellón 11. Planta 0, Madrid, 28029 Spain; 5 Dinamo Fundació, C. d’en Blanco 73, bx, Barcelona, 08028 Spain; 6 LaCol Arquitectura Cooperativa, C. Riera d’Escuder, 38, nau 2 Planta 1, Barcelona, 08028 Spain; 7 Biostatistics Support and Research Unit, Germans Trias i Pujol Research Institute and Hospital (IGTP), Barcelona, Catalonia Spain

**Keywords:** Cooperative housing, Health, Social determinants

## Abstract

**Background:**

Housing is considered a social determinant of health. In Catalonia and Spain, ensuring affordable housing is challenging and cooperative housing under a grant-of-use emerges as an alternative, challenging traditional housing models. This study aims to quantify its impact on health before and after moving to the cooperative house.

**Methods:**

A longitudinal study of individuals in cooperative housing projects in Catalonia (July 2018-April 2023) was conducted. Data, including sociodemographic, housing information, and health-related details, were collected through baseline and follow-up surveys.

**Results:**

Seventy participants (42 women, 28 men) showed positive changes in housing conditions during follow-up. Improved perceptions of health, mental health, and social support were observed. Despite limitations in sample size and short follow-up, initial findings suggest improvements in health.

**Conclusions:**

Cooperative housing under a grant-of-use in Catalonia appears promising for improving health and living conditions. Further research is warranted to explore its full potential as an alternative amid housing challenges in the region.

**Supplementary Information:**

The online version contains supplementary material available at 10.1186/s12889-024-19214-1.

## Background

Housing is a social determinant of health, and its conditions impact the physical and mental well-being of individuals through four dimensions: (1) the economic and legal aspects of housing; (2) the emotional and social meaning individuals attribute to it; (3) the physical conditions; and (4) the physical and social environment of the neighborhood where the housing is located. These dimensions are influenced by various axes of social inequality and are shaped by each country’s housing system, as well as other macroeconomic and social policies [1, 2].

In Catalonia and the Spanish context, ensuring the right to affordable housing is a major challenge. Nowadays housing is a market commodity and a financial asset rather than a fundamental right [3]. Property ownership has been promoted as the primary form of tenure, while public housing policies have lagged behind those of other European countries [4, 5].

In response to this context, alternative practices for accessing housing have emerged on the fringes of the market. An alternative model challenging the buy-versus-rent dichotomy is that of housing cooperatives with a tenure regime under a grant-of-use [6–8]. In this model, the property is collectively owned by the cooperative, and the land may be owned or leased for an extended period. Right of use as a tenure regime occurs because the cooperative, as the property owner, grants members the right to use the housing in exchange for a predetermined and stable fee outlined in an indefinite contract. Living in cooperative housing under a grant-of-use is more affordable than other types of housing. Although the initial investment required at the beginning of the project is difficult to obtain (between EUR 5,000–30,000), depending on whether the project is developed in a renovated building or newly constructed, the monthly fee is usually lower than the majority of current housing rents in the free market. It is worth mentioning that the entrance fee may be refunded if (and when) the tenant leaves the cooperative [9, 10]).

A key characteristic is that members or households cannot sell or rent the property which prevents speculative housing, the practice that considers housing as a commodity and a financial asset for financial gain, rather than a basic right, prioritizes its exchange value over its use value. The model is based on self-management and cooperative organization. Beyond tenure and collective ownership, usually individuals actively participate in housing self-promotion, design and construction, community life projects, mutual support; risk sharing, caregiving work, cooperative dynamics, and all collective needs related to housing [11–13] Additionally, involving individuals in the design process allows for the development of more sustainable housing, with improved energy efficiency, environmental commitment, and integration into the social and neighborhood fabric [14, 15].

Previous experiences in other countries, such as Denmark’s Andel model [16, 17] or the Uruguayan Federation of Housing Cooperatives for Mutual Aid (FUCVAM) [18, 19], have a long trajectory. In Catalonia, the model has evolved with 60 projects, in various stages of development (47 projects) or living together (13 projects) representing approximately 1000 housing units, according to the current census of the Housing Observatory in grant-of-use [20]. Its successful implementation is attributed to collective organization among citizens, the social sector, entities in the Social and Solidarity Economy, and the collaboration of public administrations that choose to support this housing model.

Despite the increase in projects in recent years, research on health effects is still scarce. A scientific review of alternative housing models revealed few studies directly analysing health effects, and the existing studies often have low methodological quality. Health effects are primarily explained through psychosocial determinants, such as a greater sense of community, increased social support, and greater physical, emotional, and economic security [21]. A study in Catalonia has demonstrated a positive relationship between cooperative housing under a grant-of-use and the health and quality of life of individuals. The results indicate that the model improves people’s health primarily through the benefits of sharing daily life, pooling risks and caregiving work, and the security provided by a long-term grant-of-use [9]. However, there are no longitudinal studies analysing the health effects through the process of accessing a cooperative house.

With the intention of continuing to contribute to scientific research on the relationship between the cooperative housing model and health, this study aims to quantify the impact on the health of people living in cooperative housing projects under a grant-of-use in Catalonia before and after moving to the cooperative house.

## Methods

A longitudinal study was conducted based on a dynamic cohort of individuals participating in cooperative housing projects under a grant-of-use in Catalonia. The study population included individuals participating in such projects between July 2018 and April 2023. The primary source of information was the Health and Well-being Survey designed and conducted within the project “Impact on the health of cooperative housing with a right of use” by the Housing team of the Public Health Agency of Barcelona (ASPB). The survey was conducted at four different points: at two baseline moments, when the person joined the cooperative and just before moving into the cooperative housing; on the other hand, follow-up surveys were conducted one year and two years after entering the cooperative housing.

The baseline and the follow-up questionnaires collected sociodemographic and socioeconomic data, information about housing, the relationship with the project, and health-related information at each point. Participants were interviewed by an expert of the housing and health group. Descriptive information about the cooperative housing project was collected through a baseline and follow-up form filled out by a project representative.

**Fig. 1:**
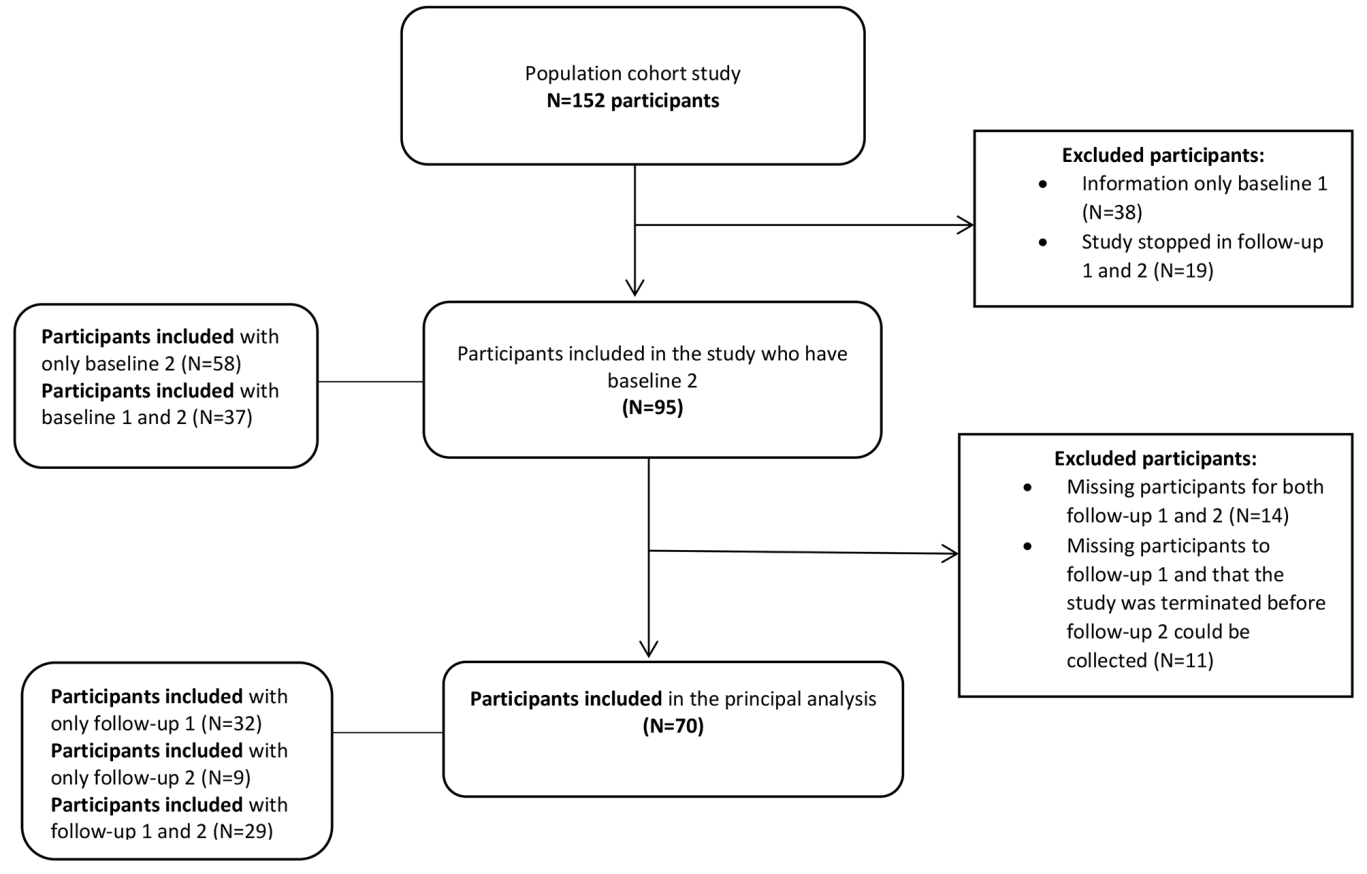
Flow chart of participants

The cohort included 152 participants from 12 cooperative housing projects. When the study started there were four cooperative housing projects ongoing. For the study, all participants who had information at baseline 2, some of them also had information at baseline 1 (except in those cases where it could not be collected because the project was already underway when the study began) and at least one follow-up moment were included, forming a sample of 70 individuals (42 women, 28 men). The flow chart of participants is available in Fig. 1.

Due to the high percentage of excluded participants, measures were taken to address possible information losses in order to maintain the integrity and validity of the study results. The characteristics of both excluded and included individuals were described and compared, revealing them to be very similar. Out of the 82 excluded participants, the average age was 43.7 (SD: 11.31), 79% had university education, and 90% were from Spain.

All participants provided informed consent, and the study was approved by the Ethics Committee of Parc de Salut Mar (ref. 2020/9372).

Sociodemographic and socioeconomic variables such as sex, age, place of birth, and level of education were included. Also, characteristics of the current housing situation such as leaks, dampness, and decay, poor building insulation, noise, capacity to maintain the suitable temperature, overcrowding, housing satisfaction and emotional attachment to the home and satisfaction with the neighbohood (Appendix 1). In this study, two health outcomes—perceived health and mental health—and one psychosocial outcome—social support—were analysed. All variables were described at baseline and follow-up.

Self-reported health was measured using the question ‘How is your health in general?’ The responses were categorized as poor and fair, good and very good, and excellent [22]. The risk of poor mental health was assessed using the 12-question version of the Goldberg Health Questionnaire (GHQ-12), categorized as poor mental health (GHQ-12 > 3) and good mental health [23]. Social support was measured using the Oslo Social Support Scale (OSSS-3) and categorized as limited social support, moderately limited social support, and strong social support [24].

Continuous variables were presented using mean and standard deviation (SD), except those with skewness or lack of normality, which were described using median and interquartile range. Categorical variables were presented with the number of cases and the total percentage. To compare baseline and follow-up, the paired t-test, Wilcoxon signed-rank sum test, and McNemar test were employed, depending on the nature of the variable. For perceived health and mental health variables, analyses were stratified by sex. Analyses were performed using the R statistical program version 4.3.0.

## Results

The average age of participants was 43.7 (SD: 12.22). 77% had university education, and 86% were from Spain. Regarding project characteristics, 90% were located in the city of Barcelona, with 77% being new construction and 23% rehabilitation. 77% made an initial contribution to the housing of less than 20,000 euros.

Regarding the housing situation, there was an improvement in all variables during the follow-up (Table 1). Leaks, dampness, and decay decreased from 37 to 4.6% (*p* < 0.001), poor building insulation decreased from 54 to 7.5% (*p* < 0.001), and the ability to maintain an adequate temperature increased from 49 to 90% (*p* < 0.001). Overcrowding increased from 1.4 to 16% (0.009). Participants at baseline had a mean housing satisfaction of 6.30 (SD: 1.92), which increased to 8.59 (SD: 1.25) at follow-up. The emotional attachment to the home had a mean of 3.66 (SD: 0.74) at baseline, which increased to 4.30 (SD: 0.58) at follow-up, and satisfaction with the neighborhood increased from 2.93 (SD: 1.04) to 3.94 (SD: 0.71) (Table 1).

**Table 1:** Description of housing and health situation before and after living in a cooperative housing under a grant-of-use

		Total *N* = 70	Baseline *N* (%)	Follow-up *N* (%)	*p*-value
**HOUSING VARIABLES**	**Leaks, dampness, and decay**	65			< 0.001
	No		41 (63.0)	62 (95.0)	
	Yes		24 (37.0)	3 (4.6)	
	**Poor building insulation**	67			< 0.001
	No		31 (46.0)	62 (93.0)	
	Yes		36 (54.0)	5 (7.5)	
	**Noise**	66			0.046
	No		45 (68.0)	56 (85.0)	
	Yes		21 (32.0)	10 (15.0)	
	**Capacity to maintain the suitable temperature**	70			< 0.001
	No		36 (51.0)	7 (10.0)	
	Yes		34 (49.0)	63 (90.0)	
	**Overcrowding**	69			0.009
	No overcrowding		68 (99.0)	58 (84.0)	
	Yes overcrowding		1 (1.4)	11 (16.0)	
	**Housing satisfaction**	70			< 0.001
	Mean and standard deviation (SD)		6.30 (1.92)	8.59 (1.25)	
	Median [Q1; Q3]		7.00 (5.25,7.75)	9.00 (8.00, 9.00)	
	**Emotional attachment to the home**	69			< 0.001
	Mean and standard deviation (SD)		3.66 (0.74)	4.30 (0.58)	
	Median [Q1; Q3]		3.83 (3.33, 4.00)	4.33 (4.17, 4.67)	
	**Satisfaction with the neighborhood**	69			< 0.001
	Mean and standard deviation (SD)		2.93 (1.04)	3.94 (0.71)	
	Median [Q1; Q3]		2.67 (2.17, 3.83)	4.00 (3.67, 4.50)	
**HEALTH VARIABLES**	**Perceived health**	69			0.474
	Poor and fair		14 (20.3)	13 (18.8)	
	Good		28 (40.6)	24 (34.8)	
	Very good and excellent		27 (39.1)	32 (46.4)	
	**Mental health**	63			0.23
	Poor mental health		20 (32.0)	13 (21.0)	
	Good mental health **Gender-stratified perceived health**		43 (68.0)	50 (79.0)	
	**Women**	41			0.911
	Poor and fair		9 (22.0	9 (22.0)	
	Good		18 (43.9)	19 (46.3)	
	Very good and excellent		14 (34.1)	13 (31.7)	
	**Men**	28			0.222
	Poor and fair		5 (17.9)	4 (14.3)	
	Good		10 (35.7)	5 (17.9)	
	Very good and excellent		13 (46.4)	19 (67.9)	
	**Gender-stratified mental health**				
	**Women**	36			0.752
	Poor mental health		12 (33.0)	10 (28.0)	
	Good mental health		24 (67.0)	26 (72.0)	
	**Men**	27			0.228
	Poor mental health		8 (30.0)	3 (11.0)	
	Good mental health		19 (70.0)	24 (89.0)	
	**Social support**	70			0.052
	Limited social support		10 (14.3)	4 (5.7)	
	Moderately limited social support		36 (51.4)	33 (47.1)	
	Strong social support		24 (34.3)	33 (47.1)	

Regarding perceived health, men with very good and excellent perceived health showed the highest percentage increase in the follow-up (from 46.4 to 67.9%). For women, there was an improvement in those with good perceived health before entering cooperative housing (from 43.9 to 46.3%) although these results were not statistically significative (Table 1). Additionally, there was an improvement in mental health, with men reporting a higher percentage of good mental health at follow-up (from 70.0 to 89.0%) compared to women (from 67.0 to 72.0%). The results were not statistically significant (Table 1).

Low and moderate social support decreased during the follow-up (from 14.0 to 5.7% and from 51.0 to 47.0%, respectively), while strong social support increased (from 34.0 to 47.0%) (*p* < 0.052) (Table 1).

## Discussion

Our results illustrate how the housing situation significantly improves when individuals move into a cooperative housing unit. Moreover, there was an observed enhancement in perceived health and mental well-being, especially among men, although the effects were not statistically significant. Social support also showed an improvement, although the results were on the verge of statistical significance.

Previous studies have highlighted how housing conditions affects people’s health. Physical conditions such as inability to maintain adequate home temperature (energy poverty), exposure to external noise, overcrowding, humidity, and mold have effects on mental health, increasing stress, anxiety, sleep disturbances, and physical health issues, including respiratory conditions, allergic reactions, and a higher risk of infectious diseases [25, 26]. Our results demonstrate how these housing conditions improve when individuals move into cooperative housing. This can be explained by the fact that in most cooperative projects, the cooperative have a strong involvement during the design of the project and sustainable design of the building, sharing goods and resources, community support and training initiatives [14]. However, our results revealed an increase in overcrowding when individuals moved into a cooperative. A key characteristic of this model is the intentionality of sharing daily life and having communal spaces. This often results in smaller private spaces in most projects, as areas such as the kitchen, laundry, and multipurpose rooms are designated for communal use. Therefore, for future research, it will be necessary to consider measuring this variable, taking into account both shared and private spaces [9].

On the other hand, there is evidence that links the lack of affordability to health problems [27]. In this regard, our results show that 77% of the participants contributed an entry fee to the housing lower than the market rate, suggesting that the model is facilitating access to housing and, consequently, may prevent health issues. Similarly, various studies explain how satisfaction with housing is associated with a better self-rated health status. Moreover, the perception of insecurity in the neighborhood can lead to a decrease in time spent outside the home, physical activity, and social relations. Our results demonstrate how individuals experience an increase in satisfaction with housing and the neighborhood when living in cooperative housing [28]. This can be explained by the self-organization of the cooperative and the collective decision-making process that fosters a sense of belonging and community [12, 29, 30]. The nature of the model leads to increased interaction among people, as, in most cases, they share their daily lives, assisting in care and other aspects. This aligns with the findings in our study regarding the increase in social support, a psychosocial factor that enhances health.

The main limitation of this study is the sample size obtained. The small number of cases in this study does not provide sufficient statistical power to conduct more complex and in-depth analyses, such as developing multivariate techniques to understand the role of potential confounding variables, considering the existing interaction between housing and other determinants. However, it is necessary to mention that cooperative housing is a new experience in Catalonia and therefore it is not possible to widen the sample size. In addition, it is difficult to improve health status in a short follow-up time, particularly in an entry population with a majority in good health. However, due to the lack of evidence of the impact of this model, these first results are very important.

## Conclusions

In conclusion, to the best of our knowledge, our research presents one of the first experiences in our context that evaluates the housing conditions and health impacts of participating in cooperative housing under a grant-of-use model. The findings suggest that cooperative housing under a grant-of-use model in Catalonia can improve the housing conditions and the people’s health. Further research is needed to investigate the relationship between cooperative housing under a grant-of -use and people’s health, as accessing housing in the region is becoming increasingly challenging, and the model can provide an alternative to the current lack of housing availability providing security in the tenure regime thanks to a stable quota over time through an indefinite contract. This study provides a basis for further exploration and research in this innovative housing model.

### Electronic supplementary material

Below is the link to the electronic supplementary material.

Supplementary Material 1

## Data Availability

No datasets were generated or analysed during the current study.
