# Supplementary material for: Cooperative housing under a grant-of-use in Catalonia and health: pre-post analysis
Source: BMC Public Health. 2024 Jul 9;24:1835. doi: 10.1186/s12889-024-19214-1 (PMC11234659; doi:10.1186/s12889-024-19214-1)
Supplement: Supplementary file 1 — Supplementary Material 1 [file 12889_2024_19214_MOESM1_ESM.docx]

**Appendix 1. Housing situation: Survey questions and their study variables**

**Leaks, dampness, and decay**

Do you have problems with leaks, dampness in walls, floors, ceilings or foundations, or rotting of floors, window frames, or doors in your home?

Dichotomous variable: (Yes/No)

**Noise**

Has the noise in the house (with windows closed) prevented or made it difficult for you to sleep?

Dichotomous variable: (Yes/No)

**Capacity to maintain the suitable temperature**

- Can you afford to keep your home at a comfortable temperature during the cold months?

Dichotomous variable: (Yes/No)

- Can you afford to maintain your home at a comfortable temperature during the warm months?

Dichotomous variable: (Yes/No)

**Housing satisfaction**

Could you please tell me how satisfied you are with your home, using a scale of 1 to 10 where 1 means 'very dissatisfied' and 10 means 'very satisfied'?

Continuous variable:

To present variable in table 1, the "housing satisfaction" variable, the mean and median of the responses obtained on the 0-10 scale were calculated.

**Emotional attachment to the home**

To what extent do you agree or disagree with the following statements, using a scale of 1 to 5 where 1 means 'strongly disagree' and 5 means 'strongly agree'?

- 1. At home, I feel like I have privacy.
  2. I can do what I want, when I want at my house
  3. Most people would like a home like mine
  4. I feel like I am in control at my home.
  5. At home, I feel safe
  6. My home expresses my personality and values

Ordinal categorical variable:

To present the variable in Table 1, the mean and median of the responses obtained on the 1-5 scale of all categories for the variable "Emotional attachment to the home" were calculated.

**Satisfaction with the neighborhood**

To what extent do you agree or disagree with the following statements about your neighborhood, using a scale of 1 to 5 where 1 means 'strongly disagree' and 5 means 'strongly agree'?

1. I am attracted to living in this neighbourhood
2. I feel like I belong in this neighbourhood
3. If given the opportunity, I would like to move to a different neighborhood.
4. I plan to continue living in this neighborhood for many years.
5. I like to see myself as similar to the people who live in this neighbourhood
6. Living in this neighborhood gives me a sense of community
7. In general, I think this is a good place to raise children

Ordinal categorical variable:

To present the variable in Table 1, the mean and median of the responses obtained on the 1-5 scale of all categories for the variable "Satisfaction with the neighborhood" were calculated.

**Overcrowding**

How many rooms does the house have? (Counting bedrooms, living room, dining room, and kitchen but NOT bathrooms) and number of people in the household unit

Dichotomous variable: (Yes/No)

- 1. Equal or more people than rooms= yes overcrowding
  2. Less people than rooms=no overcrowding
